# The History of Baculovirology in Africa

**DOI:** 10.3390/v15071519

**Published:** 2023-07-07

**Authors:** Sean Moore, Michael Jukes

**Affiliations:** 1 Citrus Research International, P.O. Box 5095, Walmer, Gqeberha 6065, South Africa; 2 Centre for Biological Control, Department of Zoology and Entomology, Rhodes University, P.O. Box 94, Makhanda 6140, South Africa; m.jukes@ru.ac.za; 3 Department of Biochemistry and Microbiology, Rhodes University, P.O. Box 94, Makhanda 6140, South Africa

**Keywords:** baculoviruses, biopesticides, Africa, entomopathogenic viruses

## Abstract

Baculovirology has been studied on the African continent for the development of insect virus-based biopesticides and, to a much lesser extent, vaccine production and delivery, since the 1960s. In this review, we focus only on baculoviruses as biopesticides for agricultural pests in Africa. At least 11 species of baculovirus have been discovered or studied on the African continent, some with several distinct isolates, with the objective in most cases being the development of a biopesticide. These include the nucleopolyhedroviruses of *Helicoverpa armigera*, *Cryptophlebia peltastica*, *Spodoptera exempta*, *Spodoptera frugiperda*, *Spodoptera littoralis*, and *Maruca vitrata*, as well as the granuloviruses of *Cydia pomonella*, *Plutella xylostella*, *Thaumatotibia* (*Cryptophlebia*) *leucotreta*, *Choristoneura occidentalis*, and *Phthorimaea operculella*. Eleven different baculovirus-based biopesticides are recorded as being registered and commercially available on the African continent. Baculoviruses are recorded to have been isolated, researched, utilised in field trials, and/or commercially deployed as biopesticides in at least 13 different African countries. Baculovirus research is ongoing in Africa, and researchers are confident that further novel species and isolates will be discovered, to the benefit of environmentally responsible agricultural pest management, not only in Africa but also elsewhere.

## 1. Introduction

*Baculoviridae* is a family of double-stranded DNA (dsDNA) viruses with more than 90 species divided into four genera: the alpha-, beta-, delta-, and gammabaculoviruses [1,2]. Baculovirus species are classified according to a new binomial naming system, while the common names and abbreviations have not changed [3]. The genome comprises a single circular dsDNA molecule with a wide range of lengths. The smallest genomes are around 80 Kbp, as seen in Neodiprion lecontei nucleopolyhedrovirus (*Gammabaculovirus nelecontei*) at 81,755 bp and 89 open reading frames (ORFs) [2]. The largest genomes are around 180 Kbp, such as in Xestia c-nigrum granulovirus (*Betabaculovirus xecnigri*) at 178,733 bp containing 181 ORFs [4]. *Baculoviridae* viruses are characterised by the formation of occlusion bodies (OBs), with members falling into two morphological groupings. Nucleopolyhedroviruses (NPVs) form large OBs, referred to as polyhedra, which range from 0.6 to 2 µM. Granuloviruses (GVs) form smaller OBs ranging from 0.2 to 0.4 µM and are referred to as granules [5]. The formation of OBs enables baculoviruses to persist under normal environmental conditions for extended periods of time. The structural characteristics of baculoviruses have been extensively studied and are reviewed in detail in several publications (see, for example, [5,6]. Baculoviruses have a broad host range, infecting the larval stages of insects in the orders Hymenoptera, Diptera, and Lepidoptera [1].

**Figure 1:**
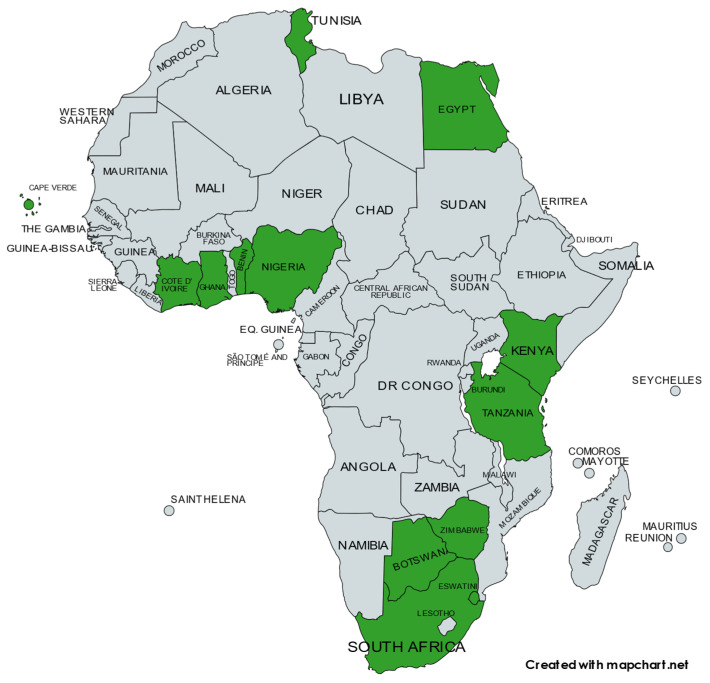
Overview of African countries where baculoviruses have been isolated, researched, utilised in field trials, and/or commercially deployed as biopesticides.

The structural and biological characteristics of baculoviruses make them well suited for use as biological pesticides for the control of lepidopteran pests [7,8,9,10]. Over the past several decades, multiple baculovirus species have been tested, formulated, and sold as commercial biopesticides in various regions of the world. These regions primarily include South America, North America, Europe, and Asia [11,12,13,14]. As with elsewhere in the world, baculoviruses have been investigated as vaccine production and delivery systems in Africa. This specifically pertains to the development of a vaccine for the African horse sickness virus, using a baculovirus expression system [15,16,17,18]. However, challenges with production yields [19], the need for repeated boost inoculations [20], and the use of potent adjuvants to enhance immunogenicity [21] have limited the usefulness and application of this type of vaccine. Consequently, in this review, we chose to focus on the research and development of baculoviruses into biopesticides for the control of important pests across the African continent. At least 11 baculovirus species have been or are being investigated for use as biopesticides in African countries, as shown in Figure 1. These investigations are broad and include the isolation and identification of novel species, the evaluation of biological activity under laboratory conditions, the determination of efficacy under field conditions, and the formulation of baculoviruses as active ingredients in commercial products for large-scale agricultural applications.

## 2. Baculoviruses

### 2.1. Alphabaculovirus Helarmigerae, Helicoverpa armigera Nucleopolyhedrovirus (HearNPV)

*Helicoverpa armigera* (Hübner) (Noctuidae: Lepidoptera) has previously been ranked as the most important lepidopteran pest in South Africa [22,23]. Whether this is still so or not is questionable; nonetheless, it remains a very important and potentially destructive polyphagous pest on the continent. The first report of the occurrence of HearNPV in Africa was by Whitlock [24], who documented the symptomatology, not only of NPV but also a GV of the same insect, enabling the identification of the causative agent, based purely on symptoms of infection. However, the viruses were identified using electron microscopy. In laboratory bioassays, the LT_50_ with HearNPV was 5–8 days, whereas for HearGV, it was 17–25 days, depending on the concentration applied [25]. Furthermore, GV-infected larvae could continue feeding for up to 35 days, precluding any potential for use in a biopesticide [24]. Whitlock [26] also identified a degree of maturation immunity (from one instar to the next) to HearNPV, now a well-known phenomenon.

Roome [27] was the first to explore the efficacy of HearNPV in field trials. This he did against *H. armigera* on sorghum and cotton in Botswana. He found that a local isolate of HearNPV was as effective as the chemical insecticide, carbaryl, and more effective than commercial preparations of Helicoverpa zea NPV (HzNPV), *Alphabaculovirus hezeae*, and *Bacillus thuringiensis* var *kurstaki* (Btk), in reducing damage on both crops. The addition of molasses enabled a halving of the concentration of HearNPV applied, without any loss of efficacy. Contrary to expectations, spraying in the evening was no more effective than spraying in the day, possibly because the virus was protected against UV irradiation in the parts of plants where the larvae fed.

More recently, Moore et al. [28] conducted field trials against *H. armigera* on citrus in South Africa. Globally, *Helicoverpa armigera* is generally not recognised as a pest of citrus. However, in South Africa, the infestation of blossoms and setting fruitlets is an annual occurrence, often requiring spraying if the intervention threshold is surpassed [29]. Trials were initially conducted on potted tomato plants within a greenhouse, in order to obtain guidance on what concentrations to use in the field. In two field trials on Navel oranges, a concentration of 7.26 × 10^5^ OBs/mL (and a couple of higher concentrations) resulted in a 100% reduction in *H. armigera* infestation within 14 days or longer. Damage to fruit was reduced by up to 84%, and rejection for export was reduced by up to 96%. These results were better than those achieved with Btk, Neem oil, and various organophosphates in the same trials. This work led to the development of the first commercially available HearNPV product in Africa, namely Helicovir (River Bioscience, Gqeberha, South Africa) [30]. Subsequently, three more products have been registered: two based on a different (non-African) isolate, Bolldex and Graboll (Andermatt Biocontrol, Grossdietwil, Switzerland), and one based on an isolate from Kenya, Helitec (Kenya Biologics, Runyenjes, Kenya) [31], initially registered in Ghana.

A few fundamental genetic and biological studies have been conducted on HearNPV in Africa. Through restriction endonuclease (REN) analysis, Ogembo [32] showed that two HearNPV isolates, one from South Africa, as originally reported by Whitlock [24], and one from Kenya, differed genetically. Ogembo et al. [33] went on to show in a dose–response study that both isolates had identical virulence to that of a commercial HzNPV product, Gemstar, against first- and second-instar *H. armigera*, but that the HearNPV isolates were more virulent than Gemstar against the third and fourth instars. Ultimately, the South African isolate was the most virulent, including having the shortest time to death in time–response assays. Ogembo et al. [34] expanded the study to include several additional isolates from Kenya and isolates from Zimbabwe and Thailand. Of the 162 clones isolated, 25 were shown to be unique, using REN. Finally, the virulence of several of the clones was compared, identifying the most promising candidate for biocontrol of *H. armigera*. Mtambanengwe [35] conducted full-genome sequencing with two novel isolates of HearNPV, collected from the Eastern Cape and KwaZulu-Natal provinces in South Africa, demonstrating their uniqueness in comparison with isolates described in studies from elsewhere.

Baillie and Bouwer [36] used next-generation sequencing (NGS) and denaturing gradient gel electrophoresis (DGGE) to identify the genetic differences between seven geographically distinct HearNPV isolates from South Africa. Depending on the gene, NGS identified between 31% and 35% of SNPs that were non-synonymous and may thus affect the biological function of the encoded proteins and therefore also the virulence of different virus isolates. Baillie and Bouwer [37] also found that inoculation dose with HearNPV against neonate *H. armigera* in bioassays significantly affected the genetic diversity of harvested virus thereafter.

Grant and Bouwer [38] demonstrated that not only differences in virulence occur between different HearNPV isolates but also different *H. armigera* populations respond differently to the same isolate. For example, although median lethal doses (LD_50_) did not differ significantly between two field-collected and one laboratory-reared population, the median lethal time (LT_50_) of the laboratory population was significantly shorter than the LT_50_ of the field populations. Bouwer et al. [39] also showed that the larvae surviving sublethal doses (LD_25_ an LD_75_) had significantly higher metabolic rates (respiration rates, measured as the rate of CO_2_ production) than that of untreated larvae. The authors postulated that this was due to a combination of viral replication, organ damage, and an energy-intensive induced cellular immune response.

### 2.2. Betabaculovirus Cypomonellae, Cydia pomonella Granulovirus (CpGV)

*Cydia pomonella* (Linnaeus) (Lepidoptera: Tortricidae), more commonly known as the codling moth, is a worldwide pest of apple, walnut, and pear. Biological control has long been implemented against *C. pomonella*, which includes entomopathogenic fungi, viruses, nematodes, bacteria, and microsporidia [40]. The betabaculovirus Cydia pomonella granulovirus (CpGV) is a highly efficacious and selective virus, which has been produced as a commercial product in Europe and North America and utilised globally for the control of *C. pomonella* since the early 1990s [41,42]. Two commercial products, Carpovirusine^®^ (Arysta LifeScience, Noguères, France) and Madex^®^ (Andermatt-Biocontrol AG, Grossdietwil, Switzerland), both of which are formulated with the Mexican CpGV isolate, are utilised in South Africa for the control of *C. pomonella* [43]. However, the continued use of CpGV in commercial products in Europe and the USA has led to the development of multiple types of resistance in *C. pomonella* populations in these regions [44,45,46,47,48,49,50]. In response, novel isolates have been continually formulated into new commercial products for use against these resistant populations.

While no resistance has yet been observed in South African populations of *C. pomonella*, a study was conducted by Motsoeneng et al. [51] to search for novel isolates that could be utilised should the need arise, leading to the isolation of CpGV-SA. The genetic analysis of CpGV-SA indicated this isolate to be unique from previously identified isolates, harbouring many unique single nucleotide polymorphisms (SNPs) [51]. Furthermore, the biological activity of CrleGV-SA and CpGV-M against *C. pomonella* was determined and compared. These isolates were found to have similar median lethal concentrations, LC_50_ and LC_90_ values at 1.6 × 10^3^ and 1.2 × 10^5^ OBs/mL for CpGV-SA and 3.1 × 10^3^ and 2.8 × 10^5^ OBs/mL for CpGV-M, respectively [52]. The LT_50_ for CpGV-SA and CpGV-M against *C. pomonella* were also determined to be 135 and 136 h, respectively, with each isolate applied at concentrations equal to their respective LC_90_ [52]. These results suggest that CpGV-SA could be developed into a commercial biopesticide and utilised as an alternative to or interchangeably with existing commercial biopesticides based on CpGV-M, both in South Africa and in other African countries where *C. pomonella* is problematic.

### 2.3. Betabaculovirus Pluxylostellae, Plutella xylostella Granulovirus (PlxyGV)

Plutella xylostella granulovirus (PlxyGV), a betabaculovirus that infects the diamondback moth, *Plutella xylostella* (L.) (Lepidoptera: Plutellidae), was first discovered in 1970 by Asayama and Osaki [53] from diseased larvae in Japan. Since then, additional isolates of PlxyGV were identified from several countries, including China, Taiwan, India, Malaysia, and of particular interest Kenya and South Africa. In field surveys conducted by Parnell et al. [54], 127 diseased *P. xylostella* cadavers collected from eight farms surrounding Nairobi, Kenya, were examined for the presence of PlxyGV. Of these, 95 cadavers were observed to be suffering from PlxyGV infections, from which 14 isolates with unique REN profile patterns were distinguished. Subsequently, laboratory and small-plot field trials were conducted by Grzywacz et al. [55] using several of these isolates, to evaluate their efficacy against *P. xylostella*. Dose–response bioassays in the laboratory demonstrated an LC_50_ ranging from 3.95 × 10^7^ OBs/mL in the PlxyGV-Nya-40 isolate to 2.36 × 10^6^ OBs/mL in the PlxyGV-Nya-01 isolate, compared with the LC_50_ of the Taiwanese reference isolate PlxyGV-Tw at 1.55 × 10^7^ OBs/mL. Small-plot field trials were conducted using the PlxyGV-Nya-01 isolate on Kale, at both high (3.0 × 10^14^ OBs/ha) and low (3.0 × 10^13^ OBs/ha) application rates and compared against an unsprayed control and a chemical insecticide treatment [55]. Damage to crops by DBM was reduced below that seen in either the chemical insecticide treatment or untreated controls for both PlxyGV application rates.

Field surveys across 92 sites in Benin were conducted in 2001, in which more than 5000 larvae were sampled and screened for PlxyGV [56]. PlxyGV was not detected in any of these West African survey sites, resulting in the importation of the Kenyan isolate PlxyGV-Nya-01 into Benin for evaluation. Dose–response assays conducted using the Kenyan isolate against the Benin *P. xylostella* population resulted in an LC_50_ of 1.15 × 10^5^ and 2.40 × 10^6^ OBs/mL for the second and third instars, respectively. The LC_50_ was reported to be considerably lower in the Benin population than that measured against the Kenyan population, with Cherry et al. [56] hypothesising major differences in prior exposure to PlxyGV by these different geographic populations as a contributing factor, as evidenced by the complete lack of PlxyGV identified during these field surveys.

Abdulkadir et al. [57] isolated a South African isolate, PlxyGV-SA, from *P. xylostella* larvae collected from a field site in the Eastern Cape Province, South Africa. Surface dose bioassays of PlxyGV-SA against neonate *P. xylostella* larvae resulted in LC_50_ and LC_90_ values of 3.56 × 10^5^ and 1.14 × 10^7^ OBs/mL, respectively [58]. The LT_50_ for PlxyGV-SA was measured to be 5.3 days, which is comparable to the 4.9 days recorded for the PlxyGV-Tw isolate against neonate *P. xylostella* [58,59]. To date, the complete genome sequences of nine PlxyGV isolates have been determined and uploaded to GenBank (NCBI), of which PlxyGV-SA is the only African isolate [60]. The high degree of isolate variation identified in Kenya by Parnell et al. [54] led to the suggestion that there has been a long association between PlxyGV and DBM in Africa. With only a single African genome sequence currently available and a multitude of African isolates already identified, much of the genetic diversity among PlxyGV isolates in Africa remains untouched and unknown. Furthermore, PlxyGV is yet to be developed into a commercial biopesticide, likely due to the lack of an artificial diet, which is required for the mass rearing of *P. xylostella* larvae.

### 2.4. Betabaculovirus Cryleucotretae, Cryptophlebia leucotreta Granulovirus (CrleGV)

The false codling moth, *Thaumatotibia leucotreta* (Meyrick) (Lepidoptera: Tortricidae), is an important agricultural pest in Africa south of the Sahara [61,62]. Although it is capable of causing economic losses in citrus fruit (other than lemons and limes [63]), peaches, peppers, pomegranates, and some other crops, its main status is phytosanitary in nature, due to its endemism to Africa. A range of control options have been developed to manage the pest [64,65,66,67], one of which is the Cryptophlebia leucotreta granulovirus (CrleGV) [68,69,70]. The genus of the host was previously *Cryptophlebia*, hence the name of the virus, but the host name was changed to *Thaumatotibia* in the late 1990s [71].

The first discovery of CrleGV was from infected larvae found in the Ivory Coast in the 1960s [72]. It was found that if the CrleGV contamination of *T. leucotreta* larvae was not curtailed in a rearing facility, this could lead to the collapse of the culture [73]. Incidentally, the authors also noted a cypovirus (CPV) infection in the same laboratory culture. A second CrleGV isolate was reported in diseased larvae from Cape Verde [74]. The occurrence of a third isolate was reported from a laboratory culture of *T. leucotreta* in South Africa in 1980 [75]. Fritsch and Huber [76], Fritsch [77], and Jehle et al. [78] demonstrated, through REN analysis, that the three isolates were genetically distinct. Jehle et al. [78] constructed a restriction fragment map covering almost the entire genome of the Cape Verde isolate of CrleGV, identifying the position of the granulin gene through cross-hybridisation with granulin coding fragments of CpGV [79]. Moore [68] described the discovery and development of an additional novel CrleGV isolate, found infecting *T. leucotreta* larvae in a mass-rearing facility in the Western Cape Province of South Africa. This isolate was genetically characterised by Singh et al. [80]. Goble [81] further genetically characterised this same CrleGV isolate, comparing it with another South African isolate. Restriction analysis and partial amplifications of the *granulin* and *ecdysteroid UDP-glucosyltransferase (egt)* genes revealed 99% and 98% nucleotide identities, respectively, between the two isolates. Most recently, Opoku-Debrah et al. [82] succeeded in inducing outbreaks of CrleGV in five geographically distinct *T. leucotreta* laboratory cultures through the overcrowding of larvae. This led to the isolation and genetic characterisation of five novel South African CrleGV isolates. The single REN analysis of viral DNA and partial sequencing of *granulin* and *egt* genes and multiple alignments of nucleotide sequences were used to demonstrate these differences, leading to a proposal for two phylogenetic CrleGV-SA groups [82].

In addition to the genetic characterisation studies, extensive biological assays have been conducted. In dose–response assays with the Cape Verde CrleGV isolate (CrleGV-CV) and five CV clones, Fritsch [77] determined that the LC_50_ ranged from 9.38 × 10^3^ to 1.86 × 10^7^ OBs/mL. Moore et al. [70] reported surface inoculation dose–response, time–response, and detached fruit bioassays against *T. leucotreta* neonate larvae. LC_50_ and LC_90_ values were estimated as 4.09 × 10^3^ and 1.18 × 10^5^ OBs/mL, respectively. LT_50_ and LT_90_ values were estimated to be 4 days 22 h and 7 days 8 h, respectively. Opoku-Debrah et al. [83] tested the virulence of five geographically distinct and two commercial isolates of CrleGV against larvae from five *T. leucotreta* laboratory cultures of different regional origins, in laboratory bioassays. They demonstrated that virulence is a very specific relationship between host and pathogen, showing that certain isolates were significantly more or less virulent against certain regionally distinct host populations. Consequently, it could not be claimed that any particular isolate was more or less virulent, without contextualising it by stating against which population the comparison was conducted.

The first use of CrleGV for the control of *T. leucotreta* was in Cape Verde in the 1980s, where one small-scale field trial was reported on citrus and Spanish pepper [84]. Virus concentrations of 10^8^ and 10^9^ OBs/mL were used, applied with skimmed milk powder and a wetting agent. Damage by *T. leucotreta* was reduced by 77% in citrus and 65% in Spanish pepper. Moore [68], Moore et al. [69], Kirkman [85], and Moore et al. [86] reported more than 50 field trials with CrleGV against *T. leucotreta* on citrus in South Africa, spanning from 2000 to 2015, the earlier trials leading to the registration of the first commercially produced CrleGV product [69]. In a representative sample of 13 field trials, Moore et al. [86] reported reducing *T. leucotreta* infestation of citrus fruit by between 30% and 92%, in field trials with CrleGV. Efficacy was shown to persist at a level of 70% for up to 17 weeks after the application of CrleGV. The addition of molasses substantially and sometimes significantly enhanced efficacy. It was also established that CrleGV should not be applied at less than ~ 2 × 10^13^ OBs/ha in order to avoid compromised efficacy [86]. In 2003, the first CrleGV product was registered in South Africa for the control of *T. leucotreta* on citrus, avocadoes, grapes, and other crops, namely Cryptogran (River Bioscience, Gqeberha, South Africa) [69,87]. Subsequently, another two products have been registered and are commercially available for use on a range of susceptible crops, i.e., Cryptex and Gratham (both Andermatt Biocontrol, Grossdietwil, Switzerland) [88].

### 2.5. Alphabaculovirus Crypeltasicae, Cryptophlebia peltastica Nucleopolyhedrovirus (CrpeNPV)

*Cryptophlebia peltastica* (Meyrick) (Lepidoptera: Tortricidae), more commonly known as the litchi moth, is an important pest of litchi (*Litchi chinensis* Sonnerat) in several sub-Saharan and Indian Ocean countries such as Mauritius, South Africa, and Réunion Island [89,90,91,92]. Marsberg et al. [93] initiated a laboratory culture of litchi moth, maintained on *T. leucotreta* artificial diet [94]. Diseased larvae were collected and examined for the presence of baculovirus infection. This resulted in the discovery of a novel nucleopolyhedrovirus, which was subsequently genetically and biologically characterised. The virus, Cryptophlebia peltastica nucleopolyhedrovirus (CrpeNPV), was examined using transmission electron microscopy, which revealed the presence of polyhedral occlusion bodies with numerous singly enveloped nucleocapsids. The phylogenetic analysis of CrpeNPV based on *late expression factor 8* (*lef-8*), *lef-9*, and *polyhedrin* sequence data confirmed the isolate to be a novel group two alphabaculovirus, with a genome length of 115,728 bp encompassing 126 ORFs (Marsberg et al. 2018). The biological activity of CrpeNPV in terms of median lethal concentration and time was evaluated against its homologous host *C. peltastica*. An LC_50_ and LC_90_ of 6.46 × 10^3^ and 2.46 × 10^5^ OBs/mL and LT_50_ and LT_90_ values of 76.32 and 93.49 h were measured, respectively [93].

As discussed above, *C. pomonella* is a serious pest worldwide. The development of several types of resistance toward existing biopesticides formulated with different CpGV isolates has created additional challenges for control strategies in Europe and the USA. A study by Wennmann et al. [95] examined the biological activity of CrpeNPV against several laboratory-reared strains of *C. pomonella*, each of which exhibited different types of resistance to CpGV infection. These included CpRR1, which exhibited type I resistance; CpR5M, which exhibited type II resistance; CpRGO, which exhibited type III resistance; and a susceptible strain CpS. Each resistance type reduces *C. pomonella* susceptibility to certain CpGV isolates, such as in CpRR1, which exhibits reduced susceptibility to CpGV-M, or CpRGO, which exhibits reduced susceptibility to CpGV-M and CpGV-S [44,49,50]. Each population was tested against CrpeNPV and two CpGV isolates, CpGV-M and CpGV-E2, the latter of which remains infectious to all these *C. pomonella* strains. CrpeNPV was shown to be highly infectious to both susceptible and CpGV-resistant *C. pomonella* strains, with biological activity similar to the resistance-breaking isolate CpGV-E2. CrpeNPV is currently undergoing registration as a biopesticide for use in Europe for the control of *C. pomonella* and *Grapholita molesta* (Busck) (Lepidoptera: Tortricidae) on pome fruit and stone fruit. It was also recently registered in South Africa for use against *C. pomonella* on pome fruit, *T. leucotreta* and *T. batrachopa* on macadamias, and *T. leucotreta* and *C. peltastica* on litchis (Sean Thackeray, Personal communication [96]).

### 2.6. Alphabaculovirus Spexemptae, Spodoptera exempta Nucleopolyhedrovirus (SpexNPV)

The larval stage of the African armyworm moth, *Spodoptera exempta* (Walker) (Lepidoptera: Noctuidae), is regarded as a devastating cyclical and migratory crop pest of maize, wheat, sorghum, and other staple crops in a large portion of sub-Saharan Africa [97]. The first finding of an NPV disease in the larvae of *S. exempta* was attributed to Graham [98] in Brown and Swaine [99]. The latter reported surveys and laboratory studies with the virus, concluding that its occurrence was widespread in field populations of *S. exempta*, particularly where there were pest outbreaks [99]. Odindo [100] concluded that epizootics in Kenya were highest in sites of high larval density, wide fluctuations in daily temperatures, and high humidity. In sites of high incidence, the epizootic led to larval population collapse within 3 weeks of the outbreak. Vilaplana et al. [101] examined the prevalence of covert infection of NPV in field populations of *S. exempta*, in Tanzania. They found that virtually all the insects collected in the field were positive for SpexNPV DNA, and 60% of these insects had transcriptionally active viruses. This was so, even in the absence of pest outbreaks, suggesting that SpexNPV is not only transmitted horizontally, but also vertically, at extremely high levels in field populations of *S. exempta* and can maintain a persistent infection without obvious symptoms. This was supported by the finding that similarly high levels of virus DNA and RNA were detected in an *S. exempta* culture that had been maintained in the laboratory for 5 years [101]. Subsequently, Graham et al. [102] developed a real-time quantitative polymerase chain reaction (qPCR) procedure for the specific detection and quantification of SpexNPV. They used this assay for the quantification of covert virus infection in asymptomatic larvae, concluding that viral load peaked in early instars (up to 6 days post-hatch), and decreased markedly with larval age, particularly during the final two instars.

Brown et al. [103] were the first to provide a physical map of SpexNPV, constructed using a range of restriction enzymes, and it showed a genome size of 131.89 Kbp. More recently, Redman et al. [104] cloned an isolate of SpexNPV in vivo, identifying at least 17 genetically distinct genotypes, which varied in size from approximately 115 to 153 Kbp. Subsequently, Escasa et al. [105] reported fully sequencing the genome of a SpexNPV isolate, collected in Tanzania in the 1970s, and thus very likely the same isolate as investigated by Redman et al. [104]. The genome was 129.5 Kbp.

Harrap et al. [106] established a laboratory culture of *S. exempta*, field-collected in Kenya, from which an NPV infection was isolated. Subsequently, Cherry et al. [107] developed and optimised an in vivo production system for SpexNPV. The larvae of *S. exempta* were reared in individual cells (17 × 17 × 40 mm) on an artificial diet that had been sprayed with a range of concentrations. The maximum yield obtained per larva was 3.29 × 10^9^ OB per larva after 7 days of incubation. LT_50_ values fell with an increase in dose, being as short as 5.5 days when using 1 × 10^7^ OB/cell. A virus production protocol was refined and prescribed in detail by Mushobozi et al. [108], including guidelines for the formulation and application of the virus preparation.

McKinley et al. [109] found that this virus was cross-infectious with the fall armyworm, *S. frugiperda* (Smith) (Lepidoptera: Noctuidae). However, rather than being directly pathogenic to the heterologous host, it appeared to trigger the homologous virus through stress induction. Odindo [110], through a series of dose–response and time–response bioassays with third-instar *S. exempta* and its virus, established the LD_50_ as 48 OBs per larva and the LT_50_ to range from 146 to 221 h, depending on the dosage applied.

A project managed by the Natural Resources Institute in the UK was launched in 1996 to develop and evaluate the use of SpexNPV as a biopesticide alternative to chemical control [111]. The project ran for at least a decade [112]. Field trials demonstrated that both the ground and aerial application of SpexNPV to armyworm outbreaks on pasture could initiate virus epizootics and population collapse [113,114]. SpexNPV was effective when applied at 1 x 10^12^ OB/ha if applied early during an outbreak and before larvae reached the fourth instar. Mass mortality was apparent 3–10 days after application. Efficacy in field trials in northern Tanzania, with doses as low as 1 x 10^11^ OB/ha, ranged from 22% to around 90% [115]. These differences in efficacy were attributed to the presence or absence of *Wolbachia*, which was related to an increase in the virulence of SpexNPV, contrary to most other findings with *Wolbachia* [112]. It was concluded that SpexNPV was sufficiently effective to replace a chemical pesticide strategy for controlling *S. exempta* [114]. Consequently, a large biopesticide production facility for SpexNPV was built in Arusha, Tanzania, from 2008 to 2011 [116]. Despite this, the field production of the virus was conducted instead, based on the Brazilian system for production of the Anticarsia gemmatalis NPV (AgNPV), *Alphabaculovirus angemmatalis* [117,118]. This was deemed technically feasible because of the large synchronous outbreaks of larvae at high densities that are a feature of this pest [113,114]. Unfortunately, the facility never became fully operational, despite the compelling arguments made in favour of the programme [119]. This was partly due to a subsequent decline in the pest status of *S. exempta* and the arrival of *S. frugiperda* [120], which superseded the latter in research and management efforts.

### 2.7. Alphabaculovirus Spofrugiperdae, Spodoptera frugiperda Nucleopolyhedrovirus (SfNPV)

*Spodoptera frugiperda* is native to tropical and subtropical regions of the American continents, stretching from Argentina in the south to southern Florida and Texas in the north [121,122]. It was first reported as an invader in Africa in 2016, appearing in Nigeria, Sao Tomé, Benin, and Togo [123]. It was most recently confirmed to be in more than 30 African countries, including as far south as South Africa [124]. *Spodoptera frugiperda* is highly polyphagous [125,126] but is mainly a pest of maize, followed by sorghum, cotton, sweet corn, and sugarcane [127]. It is considered a devastating pest throughout much of Africa, causing estimated yield losses in maize on the continent of USD 9.4 billion [128]. Despite this, surprisingly little research has been conducted on the Spodoptera frugiperda nucleopolyhedrovirus (SfNPV) on the continent, possibly because a notable body of research from elsewhere already exists (e.g., see [129,130,131,132,133,134,135]). Consequently, a commercial preparation of SfNPV, Fawligen (AgBiTech) was relatively rapidly registered in Africa, initially in Kenya [127]. An SfNPV isolate has also now been reported from Africa (Nigeria) and has been genetically characterised [136], showing a close relatedness to isolates described from the Americas, particularly from Brazil [137].

### 2.8. Alphabaculovirus Spolittoralis, Spodoptera littoralis Nucleopolyhedrovirus (SpliNPV)

*Spodoptera littoralis* (Boisd) (Lepidoptera: Noctuidae), the Egyptian cotton leafworm, is a polyphagous insect of important crops in Africa, Mediterranean Europe, and countries in the Middle East [138,139]. The baculovirus-based control of *S. littoralis* has a long history in Africa, with early field trials and evaluations dating back to the 1980s [140,141]. Field trials of Spodoptera littoralis nucleopolyhedrovirus (SpliNPV) were conducted by Topper et al. [140] against *S. littoralis* in Egypt, to compare formulations, spraying techniques, and virus efficacy. Although no difference was found between the spray technique and formulation, a significant reduction in leaf damage was measured in the SpliNPV treatments compared with an unsprayed control application. Large-scale field trials further showed decreased leaf damage for the SpliNPV treatment as compared to the control; however, no difference in cotton yield was observed. This lack of difference was attributed to low levels of damage, with virus treatments only applied to fields that exceeded a predetermined egg mass per ha threshold.

In field trials conducted in 1988 and 1990, Jones et al. [141] evaluated the use of a wettable powder formulation of SpliNPV for the control of *S. littoralis*. Three different virus concentrations were applied and compared against the traditional method of control, which involved the collection of egg masses by hand. The analysis of the 1988 field trial showed a significant reduction in mean leaf damage 14 days after treatment for both the egg collection and the two highest NPV concentrations as compared to the control. The NPV treatments further showed a significant reduction in mean leaf damage 14 days after spraying as compared to the control and egg collection treatments in the 1990 field trials. The highest NPV concentration maintained a significant reduction in leaf damage 21 days after application. In both the 1988 and 1990 field trials, a clear dose-dependent response was observed, with higher doses correlating with reduced leaf damage.

More recently, a study by Adel-Satter et al. [142] evaluated the resistance to different pyrethroids and biocides in several geographically distinct strains of *S. littoralis*, collected from isolated field populations in Egypt across three cotton seasons (2006, 2007, and 2008). Included among the biocides tested were several formulations of an NPV that infects *S. littoralis* (presumably SpliNPV). The results obtained showed that all field strains were highly susceptible to the NPV biocide, while high levels of resistance to pyrethroid insecticides were observed. The results from this study strongly support the use of NPV-based biocides for the control of *S. littoralis* in Egyptian cotton fields, either as a replacement to conventional insecticides or for strategic use in integrated pest management (IPM) programmes. Furthermore, research by Alfy et al. [143] investigated interactions between *Bacillus thuringiensis* subsp. *mexicanensis* (Btm) on SpliNPV, applied against *S. littoralis*. The virus was isolated from larvae collected at the Nubaria Agricultural Research Station, Egypt, with semi-field trials conducted in 2017 and 2018. The control of *S. littoralis* by SpliNPV alone and in combination with Btm was evaluated. Synergistic interactions were observed, with the combination of Btm and SpliNPV providing the highest measurement of *S. littoralis* larval mortality. Although a commercial bioinsecticide using SpliNPV has been formulated and sold under the name Littovir (Andermatt Biocontrol, Switzerland) in several European countries, the virus is yet to be developed into a product for use in African countries.

### 2.9. Betabaculovirus Choccidentalis, Choristoneura occidentalis Granulovirus (ChocGV)

The citrus leafroller, *Choristoneura occidentalis* (Walsingham) (Lepidoptera: Tortricidae), is native to South Africa but more widely distributed throughout sub-Saharan Africa, with records from Gambia, Kenya, Mozambique, Zimbabwe, Sierra Leone, Tanzania, and Zambia [144]. In South Africa, it is recognised as a pest of citrus, avocado, coffee, and various ornamentals [145,146]. It should not be confused with the North American species of the same name, *C. occidentalis* (Freeman). The citrus leafroller was moved from the genus *Archips* to *Choristoneura* by Razowski [147]. However, based on a phylogenetic study, Fagua et al. [148] recommended its transfer to the genus *Cacoecimorpha* or possibly even a reversion to *Archips* (Giovanny Fagua, Personal communication [149]).

Smith and Graça [150] reported infected *C. occidentalis* larvae from citrus in Eswatini (Swaziland). Upon electron microscopic inspection, the disease was determined to be caused by a granulovirus. However, the causative organism was only morphologically characterised, and it was therefore not determined whether this was a novel granulovirus and what its relatedness is to the more recently described *Choristoneura occidentalis* granulovirus (ChocGV), characterised and described by Escasa et al. [151] from the North American host species. Due to the disparity in host nomenclature, it is highly likely that these are two distinct baculovirus species and that the name for this African species should thus not be ChocGV. This must be resolved. No further work has been reported, and consequently, its potential for exploitation as a control agent against *C. occidentalis* must still be determined.

### 2.10. Alphabaculovirus Mavitratae, Maruca vitrata Multi-Nucleopolyhedrovirus (MaviMNPV)

The legume pod borer, *Maruca vitrata* (Fabricius) (Lepidoptera: Crambidae), is an important pest of cowpea (*Vigna unguiculata* (L.) Walp. (Fabales: Fabaceae) in West Africa. Srinivasan et al. [152] evaluated the efficacy of a Taiwanese isolate of Maruca vitrata multi-nucleopolyhedrovirus (MaviMNPV) against *M. vitrata* in semi-field caged experiments at IITA, in Benin. *Maruca vitrata* larval mortality increased by 78.8% following the application of MaviMNPV at a concentration of 2 × 10^13^ OBs/ha, 7 days after spraying, as compared to an untreated control application. The authors also noted the detection of *M. vitrata* larvae with apparent MaviMNPV symptomatology at a site 150 km from where the semi-field trials were conducted. MaviMNPV had not previously been detected or released into the open in Benin. Srinivasan et al. [152] evaluated whether the *M. vitrata* parasitoid, *Apanteles taragamae* Viereck (Hymenoptera: Braconidae), was involved in the transmission of MaviMNPV. It was found that the partial or complete body contamination of *A. taragamae* with MaviMNPV resulted in the transmission of the virus to *M. vitrata*, as compared to control treatments where the parasitoid was not contaminated.

Laboratory and field trials were further conducted by Sokame et al. [153] using the same Taiwanese isolate of MaviMNPV in Benin. Experiments evaluated the effect of combining the virus with different botanical oils against *M. vitrata*. The laboratory results showed a significant decrease in the mean survival time (MST) of *M. vitrata* when MaviMNPV was applied alone, decreasing from 14+ days in the control to 8.0 ± 2.1 and 5.0 ± 0.8 days at concentrations of 3 × 10^4^ and 5 × 10^6^ OBs/mL, respectively. The MST was further decreased to 2.0 ± 0.2 days when MaviMNPV (at a concentration of 5 × 10^6^ OBs/mL) was combined with *Azadirachta indica* (neem) oil at 0.5%. A similar synergistic interaction was observed when evaluating the percentage mortality induced by MaviMNPV, increasing from 56.7% when applied alone at a concentration of 5 × 10^6^ OBs/mL to 97.5% with the addition of 0.5% *A. indica* oil. A significant reduction in damage (30.7 ± 4.2% to 13.6 ± 1.0%) and loss (24.8 ± 3.5% to 7.2 ± 0.6%), and an increase in yield (523.0 ± 28.2 to 875.1 ± 47.8 Kg/ha) of *M. vitrata* grain was further demonstrated by Sokame et al. [153] during the first of two growing seasons in 2011 when using MaviMNPV as compared to control treatments in the field. Although MaviMNPV outperformed the botanical oils evaluated during this growing season, treatment with the chemical deltamethrin was observed to provide the greatest degree of *M. vitrata* control. The combination of MaviMNPV with *A. indica* oil did, however, significantly reduce grain loss to 1.7 ± 0.3%, which was comparable to the chemical control, indicating an additive or synergistic interaction. No statistically significant results were obtained from field trials conducted during the second growing season.

### 2.11. Betabaculovirus Phoperculellae, Phthorimaea operculella Granulovirus (PhopGV)

The baculovirus Phthorimaea operculella granulovirus (PhopGV) was discovered in Australia in 1964 [154], although the first report in Africa was by Broodryk and Pretorius [155] in South Africa. The virus is known to infect at least six species within the family Gelechiidae, some of which are important pests of solanaceous crops worldwide. These are the potato tuber moth, *Phthorimaea operculella* (Zeller); the Guatemala potato tuber moth, *Tecia solanivora* (Povolny); the tomato leaf miner, *Phthorimaea absoluta* (Meyrick); *Eurysacca quinoa* (Povolny); *Symmetrischema tangolias* (Gyen); and *Paraschema detectendum* (Povolny) [156,157,158,159,160].

Substantial genetic analyses have been conducted on PhopGV, with partial sequence data available for most isolates and complete genome sequence data available for at least 12 isolates, including two from Africa [156,159,161]. These studies have resulted in each PhopGV isolate being assigned to either one of five groups based on partial *egt* gene sequences or one of four groups based on whole-genome sequence analysis. This excludes PhopGV-SA, which did not cluster within the four groups. At least 10 African PhopGV isolates have been reported, all of which originated from infected *P. operculella* larvae [155,156,156,161,162,163,164].

The evaluation of the first known African PhopGV isolate by Broodryk and Pretorius [155] showed a significantly lower survival of *P. operculella* larvae when treated with PhopGV as compared to an untreated control application. Subsequently, field trials of PhopGV as spray and powdered (combined with magnesium silicate) formulations for the control of *P. operculella* were evaluated by Salah and Aalbu [163] in Tunisia. In their first experiment, PhopGV was applied to the soil surface as a spray at 50, 75, 88, and 94 days after the emergence of the plants, with tubers evaluated weekly from four weeks prior to harvest for *P. operculella* damage. The second experiment involved the utilisation of the powdered formulation of PhopGV, with the powder applied to elevated soil surfaces 50 and 75 days after the emergence of the plants. Damage by *P. operculella* was evaluated at harvest by assessing 100 tubers per treatment. A significant reduction in *P. operculella* tuber damage was recorded for the spray formulation at only 6% damage, as compared to a control (no spray) at 19.77% damage, and a water-only spray at 29.33% damage. The powdered formulation was not significantly different from the control (no spray), at 14.33% tuber damage recorded. The effect of these treatments on stored potatoes was further examined, with both the spray and powder formulations reducing *P. operculella* damage after 30 days of storage.

Recently, several new African PhopGV isolates were identified in diseased larvae originating from Tunisia, Kenya, and South Africa [156,161]. Ben Tiba et al. [156] characterised five isolates: three from Tunisia, one from Kenya, and one from Yemen. A discriminating dose of 1.8 × 10^5^ OBs/mL was utilised to evaluate the biological activity of all five isolates against neonate larvae from two geographic *P. operculella* populations (Italy and Tunisia) and a *P. absoluta* population. The highest and lowest mortalities were observed against the *P. absoluta* population, with PhopGV-Tu1.11 having the highest measured mean mortality at 70.3% and PhopGV-Ym14 the lowest measured mean mortality at 11.7%. The mean mortality of these isolates ranged between 34% and 60% against neonate larvae from either of the *P. operculella* populations. These results suggest that PhopGV could be utilised as a biopesticide for the control of both *P. operculella* and *P. absoluta* in Africa. The recent development of PhopGV into the biopesticide Tutavir (Andermatt Biocontrol, Switzerland) in Europe suggests that this virus has commercial potential, which may prompt further research and development in African countries.

## 3. Conclusions

The exploration and investigation of baculoviruses for development as biopesticides for the management of agricultural pests have been pursued in Africa since the 1960s, when the occurrence of a CrleGV isolate was first reported in *T. leucotreta* larvae in the Ivory Coast [72]. Since then, the occurrence of at least a further series of 10 baculovirus species has been reported in agriculturally important pests, with numerous distinct isolates of some of these species (Table 1). To date, at least 11 baculovirus products have been registered and made commercially available for use in Africa, several of these being based on African isolates and produced in Africa. However, to date, there are only two known commercial baculovirus biopesticide production facilities in Africa: River Bioscience in South Africa and Kenya Biologics in Kenya.

Africa is a continent of high biological diversity, with the estimated plant and insect diversity for the Afrotropics second only to the Neotropics [165]. Thus, the small number of baculoviruses studied on the continent and exploited for the biological control of important lepidopteran pests must be considered the tip of the iceberg. The relatively recent discovery of the first NPV in the grapholitini tribe of tortricids [93] is considered a breakthrough of seismic proportions in the world of agricultural baculovirology. The grapholitini tribe consists of several globally important agricultural pests, such as the codling moth, *C. pomonella*; the false codling moth, *T. leucotreta*; the oriental fruit moth, *G. molesta*; the litchi moth, *C. peltastica*; and a couple of species of macadamia nut borer, *T. batrachopa* and *C. ombrodelta*. This NPV, CrpeNPV, has an excitingly broad host range, being virulent against most [93], if not all of these species, including *C. pomonella* resistant to its own granulovirus, CpGV [95].

Work on baculovirology in Africa is continuing, and if it retains a large exploratory component, it is likely that in the near future, a suite of novel species and isolates will emerge. Furthermore, production costs in Africa are much lower than in industrialised countries [116]. It should therefore be possible to produce baculovirus biopesticides at a much more affordable price than is the case with those produced outside of the continent and imported into Africa, creating an opportunity both for increased local uptake and for biopesticide exports. It is critical that this endeavour be encouraged and funded across the continent, in order to prepare for the growing global move towards more environmentally sustainable means of farming, with reduced chemical inputs, driven by initiatives such as the European Green Deal and Farm to Fork Strategy [67,166].

## Figures and Tables

**Table 1:** Overview of baculovirus species researched and/or utilised in Africa for the control of important agricultural pests.

Virus	Country of Origin	Targeted Host	Activities	Field Trial Crops	Number of Isolates ^2^	Isolate References
ChocGV	Eswatini	*C. occidentalis*	LT	-	1	[150]
CpGV	South Africa	*C. pomonella*	LT, BA, BD ^1^	-	2	[43,51]
CrleGV	Cape Verde, Ivory Coast, South Africa	*T. leucotreta*	LT, BA, FT, BD	Citrus, Spanish pepper, Avocado, Grape	9	[72,74,75,80,82]
CrpeNPV	South Africa	*C. peltastica*, *C. pomonella*, *G. molesta*, *T. batrachopa*, *T. leucotreta*	LT, BA, FT, BD	Citrus, Pome fruit, Stone fruit, Macadamia, Litchi	1	[93]
HearNPV	Botswana, Kenya, Ghana, South Africa, Zimbabwe	*H. armigera*	LT, BA, FT, BD	Sorghum, Cotton, Tomato, Citrus	39	[24,27,28,32,34,35,36]
MaviMNPV	Benin	*M. vitrata*	BA, FT	Legume	1	[152]
PhopGV	Kenya, South Africa, Tunisia	*P. operculella*, *P. absoluta*	LT, BA, FT	Potato, Tomato	10	[155,156,159,161,162,163,164]
PlxyGV	Benin, Kenya, South Africa	*P. xylostella*	LT, BA, FT	Kale	15	[54,57]
SfNPV	Kenya, Nigeria	*S. frugiperda*	LT, BD ^1^	-	2	[127,136]
SpexNPV	Tanzania, Kenya	*S. exempta*, *S. frugiperda*	LT, BA, FT, BD	Pasture	2	[105,106]
SpliNPV	Egypt	*S. littoralis*	FT	Cotton	2	[140,142]

LT = laboratory testing, BA = biological assay, FT = field trials, BD = biopesticide development. ^1^ Biopesticides developed outside of Africa with non-African isolate. ^2^ Estimated isolate number based on individual reports.

## Data Availability

No new data were created or analyzed in this study. Data sharing is not applicable to this article.
